# Old subjects with sepsis in the emergency department: trend analysis of case fatality rate

**DOI:** 10.1186/s12877-019-1384-8

**Published:** 2019-12-23

**Authors:** Andrea Fabbri, Giulio Marchesini, Barbara Benazzi, Alice Morelli, Danilo Montesi, Cesare Bini, Stefano Giovanni Rizzo

**Affiliations:** 1 Emergency Department, Presidio Ospedaliero Morgagni-Pierantoni, AUSL della Romagna, via C. Forlanini 34, 47121 Forlì, FC Italy; 2 Department of Medical and Surgical Sciences, “Alma Mater” University, S. Orsola-Malpighi Hospital, Via Massarenti 9, I-40138 Bologna, Italy; 3 Department of Computer Science and Engineering, Alma Mater University, Mura Anteo Zamboni 7, 40127 Bologna, Italy; 4 Healthcare Management Unit, Presidio Ospedaliero Morgagni-Pierantoni, AUSL della Romagna, via C. Forlanini 34, 47121 Forlì, FC Italy; 5 Qatar Computing research Institute (QCRI), HBKU, Doha, Qatar

**Keywords:** Sepsis, Elderly, Case fatality rate, Risk score, Trend, Emergency department

## Abstract

**Background:**

The burden of sepsis represents a global health care problem. We aimed to assess the case fatality rate (CFR) and its predictors in subjects with sepsis admitted to a general Italian hospital from 2009 to 2016, stratified by risk score.

**Methods:**

We performed a retrospective analysis of all sepsis-related hospitalizations after Emergency Department (ED) visit in a public Italian hospital in an 8-year period. A risk score to predict CFR was computed by logistic regression analysis of selected variables in a training set (2009–2012), and then confirmed in the whole study population. A trend analysis of CFR during the study period was performed dividing patient as high-risk (upper tertile of risk score) or low-risk.

**Results:**

Two thousand four hundred ninety-two subjects were included. Over time the incidental admission rate (no. of sepsis-related admissions per 100 total admissions) increased from 4.1% (2009–2010) to 5.4% (2015–2016); *P* < 0.001, accompanied by a reduced CFR (from 38.0 to 18.4%; *P* < 0.001). A group of 10 variables (admission to intensive care unit, cardio-vascular dysfunction, HIV infection, diabetes, age ≥ 80 years, respiratory diseases, number of organ dysfunction, digestive diseases, dementia and cancer) were selected by the logistic model to predict CFR with good accuracy: AUC 0.873 [0.009]. Along the years CFR decreased from 31.8% (2009–2010) to 25.0% (2015–2016); *P* = 0.007. The relative proportion of subjects **≥**80 years (overall, 52.9% of cases) and classified as high-risk did not change along the years. CFR decreased only in low-risk subjects (from 13.3 to 5.2%; *P* < 0.001), and particularly in those aged ≥80 (from 18.2 to 6.6%; *P* = 0.003), but not in high-risk individuals (from 69.9 to 64.2%; *P* = 0.713).

**Conclusion:**

Between 2009 and 2016 the incidence of sepsis-related hospitalization increased in a general Italian hospital, with a downward trend in CFR, only limited to low-risk patients and particularly to subjects **≥**80 years.

## Introduction

The elderly population is considered to be at high-risk for sepsis, due to multiple comorbidities, frailty, repeated or prolonged hospitalizations [1, 2] with a worsening outcome. Large nationwide registries indicate that up to 60% of patients with sepsis are over 65, with a positive trend for diagnosis of 1.5% per year [3], with particular relevance in subjects ≥80 years [2]. Epidemiological studies also showed an increased incidence of diagnoses following the implementation of clinical guidelines, with decreased mortality [4].

Old patients with sepsis, compared with adults may differ in several aspects: the primary sites of infection and organ system dysfunction may be different, which may have an impact on the final outcome [5]. In the presence of an increased number of diagnoses, it is not known whether the clinical characteristics and the case fatality rate (CFR) of elderly subjects with sepsis-related hospitalizations are decreasing at a similar rate as observed in the adult population [3].Aim of the study was to evaluate the association between the main characteristics and CFR of sepsis-related hospital admissions of older subjects in a general Italian hospital, in a trend analysis between 2009 and 2016.

## Methods

### Study design

In a chart review analysis we included all adult sepsis-related hospitalizations in the District hospital of Forlì (FC), Italy from 2009 to 2016 as defined by Angus et al [6]. In ED an electronic warning system is available (Systemic Inflammatory Response syndrome - SIRS) [7] for the early detection of patients at high risk of sepsis since 2007. The final diagnosis was derived from hospital discharge codes (see below).

### Registry data

The community hospital has a total capacity of 463 beds; during the 8-year study period, over 55,000 cases were hospitalized after ED visit for surgical and medical diseases out of 170,000 admissions. The hospital database is directly connected with the General Registry Office of the District. The study was approved by the ethical committee of CEROM Romagna, Italy (2299/2019/O/OssN, January 16, 2019).

### Study population

The study included any subject with ICD9-CM code for both bacterial and fungal infections and acute organ dysfunction with a code extraction method according to the 3-rd International Consensus definition for sepsis (Sepsis-3) [8, 9]. As the study period predated the 2016 definitions [6], sepsis patients were defined by any ICD9-CM codes for both bacterial and fungal infections and acute dysfunction as previously suggested [10, 11]. In order to include all cases, we also included cases explicitly coded as severe sepsis (995.92) or septic shock (785.52), in accord with the previous definition of ED sepsis. This approach was accepted as compatible with the 3rd International Consensus definition for sepsis (SEPSIS-3) [9].All information extracted by diagnosis codes where then matched with key information recorded at ED arrival, also in cases where diagnosis of sepsis was not suspected at time of ED presentation.

### Data variables

Selected variables for the analyses were demographic characteristics, main comorbid conditions, serious infection diagnoses, and organ dysfunction diagnoses. Other key pieces of information were the mode of arrival in ED, triage vital signs, SIRS score ≥ 2, ED waiting time, ED length-of-stay, type of serious infection diagnosis codes and organ dysfunction diagnosis codes, intensive care Unit (ICU) admission.

Data abstractors identified up to 5 documented diagnoses for each patient by ICD9-CM codes. In-hospital case fatality rate (CFR, i.e., the proportion of all-cause mortality) was verified by a linked local death certificate database and considered for the prognostic model.

### Statistical analysis

The characteristics and outcomes of patients were compared across the 8-year study period. Mean value, standard deviation, median, interquartile range, number of cases, percent with 95% confidence interval was used to describe data distribution. Fisher’s exact test for categorical variables and Student t-test for continuous variables were used to compare variables between groups.

The variables tested for multivariable analyses were: age, sex, arrival by emergency medical service, SIRS at entry, location at admission (ICU vs. ordinary ward), length of ED stay, and diagnoses codes of serious infection and organ dysfunction. Associated diseases were also considered, as measured by Charlson’s Index [8] calculated on the basis of the main comorbidities, in particular diabetes mellitus (DM), chronic obstructive pulmonary disease (COPD), chronic kidney disease (CKD), history of acute heart failure (AHF), dementia, cancer, HIV infection.

A multivariable model was developed by stepwise forward analysis of factors considered significant in univariable analysis and according to clinically relevant predictors. To reduce the over fitting effect of the variables in an 8-year study period, data from the entire database were separated in two different periods for model building in the training set (2009–2012; *N* = 984) and then validated in the remaining cases. Since the analysis did not produce any difference in the ROC curve, the values of the training set were applied to the whole cohort. For the model building part of the analysis, the variables were selected on the basis of previous reports and a putative association with main outcome measures, in particular CFR. As it is recommended that covariates be introduced generously into the model, we included a large number (*N* = 27) of covariates independently of significance thresholds or other selection criteria. The full list of covariates can be found in Tables 1 and 2. The colinearity of combination of variables was tested by the variation inflation factors (<2, not significant). The accuracy of the scoring system was determined by calculating the area under the receiver operating characteristic (ROC) curve with standard error. A prognostic model was also performed to define factors associated with all cause CFR. ROC curves were compared by DeLong test.

**Table 1:** Characteristics of patients with diagnosis of sepsis in relation to age (2009–2016). Data reported as number of cases and percent and mean and [standard deviation]

Characteristics	All Cases	<80 years	≥80 years	OR (95%CI) or P value
Age group	2492	1173 (47.1)	1319 (52.9)	1.11 (1.03–1.19)
Sex (male %)	1355 (54.4)	672 (57.3)	683 (51.8)*	0.80 (0.68–0.94)
Age (years, mean [SD])	77.9 [14.6]	66.5 [12.4]	88.1 [6.8]*	< 0.001
Comorbidities				
Diabetes	727 (29.2)	300 (25.6)	427 (32.4)*	1.39 (1.17 - 1.66)
COPD	1142 (45.8)	474 (40.4)	668 (50.6)*	1.51 (1.29–1.77)
CKD	837 (33.6)	366 (31.2)	471 (35.7)*	1.22 (1.04–1.45)
AHF	885 (35.5)	358 (30.5)	538 (40.8)*	1.56 (1.33–1.85)
Dementia	750 (30.1)	213 (18.2)	537 (40.7)*	3.19 (2.66–3.82)
Cancer	1046 (42.0)	525 (44.8)	521 (39.5)*	0.81 (0.69–0.94)
HIV infection	24 (1.0)	2 (0.2)	22 (1.9)*	0.79 (0.19–0.34)
Vial Signs at ED arrival				
Body temperature C	37.6 (0.8)	37.7 (0.8)	37.5 (0.7)*	<0.001
Heart rate (beats/min)	97.2 (19.6)	98.2 (19.6)	96.3 (19.6)*	0.016
Systolic blood pressure (mmHg)	117.5 (24.0)	117.2 (23.0)	117.9 (24.8)	0.464
Respiratory rate (breaths/min)	23.1 (5.8)	22.9 (5.9)	23.2 (5.8)	0.201
qSOFA ≥ 2	2302 (92.4)	1103 (94.0)	1199 (90.9)*	0.63 (0.47–0.86)
SIRS ≥ 2	1878 (75.4)	886 (75.5)	992 (75.2)	0.98 (0.82–1.18)
Diagnosis at admission	819 (32.9)	350 (29.8)	469 (35.6)*	1.30 (1.10–1.53)

*COPD* Chronic obstructive pulmonary disease, *CKD* Chronic kidney disease, *AHF* Acute heart failure, *HIV* Human immune-deficiency virus, *qSOFA*, quick Sequential Organ Dysfunction Assessment, *SIRS* Systemic Inflammatory Response Syndrome. * significant difference vs. subjects <80 years, *P* < 0.05

**Table 2:** Characteristics of subjects with sepsis related hospitalizations, grouped by age

Variables	All Cases	<80 years	≥80 years	Odds Ratio
	*n* = 2492	*n* = 1173	*n* = 1319	(95%CI)
Serious Infection Diagnosis				
Infection/parasitic	233 (9.3)	153 (13.0)	90 (6.8)*	0.49 (0.37–0.64)
Nervous system	86 (3.5)	57 (4.9)	29 (2.2)*	0.44 (0.28–0.69)
Circulatory system	88 (3.5)	42 (3.6)	46 (3.5)	0.97 (0.64–1.49
Respiratory system	1597 (64.1)	640 (54.6)	957 (72.6)*	2.20 (1.86–2.60)
Digestive system	281 (11.3)	153 (13.0)	128 (9.7)*	0.72 (0.56–0.92)
Genitourinary system	658 (26.4)	312 (26.6)	346 (26.2)	1.01 (0.93–1.10)
Pregnancy / puerpuerium	5 (0.2)	1 (0.1)	4 (0.3)	3.60 (0.40–31.9)
Skin and subcutaneous tissue	41 (1.6)	21 (1.8)	20 (1.5)	0.84 (0.45–1.87)
Muscular-skeletal system	62 (2.5)	35 (3.0)	27 (2.0)	0.68 (0.41–1.13)
Other	84 (3.4)	41 (3.5)	43 (3.3)	0.93 (0.60–1.44)
Organ Dysfunction Diagnosis				
Cardiovascular	677 (27.2)	281 (24.0)	396 (30.0)*	1.36 (1.14–1.63)
Hematologic	75 (3.0)	46 (3.9)	29 (2.2)*	0.55 (0.44–0.88)
Hepatic	55 (2.2)	32 (2.7)	23 (1.7)	0.63 (0.37–1.09)
Neurologic	67 (2.7)	45 (3.8)	22 (1.7)*	0.42 (0.25–0.71)
Renal	433 (17.4)	197 (16.8)	236 (17.9)	1.08 (0.88–1.33)
Pulmonary	1305 (52.4)	542 (46.2)	763 (57.8)*	1.60 (1.36–1.87)

95% CI: 95% confidence intervals, * significance difference vs. subjects <80 years; *P* < 0.05

Temporal trends of incidence and outcome were calculated as the rate difference between 2009 and 2016. Trends were tested by Poisson distribution analysis and represented by line graphs as mean with 95% confidence intervals.

In the analyses subjects were stratified by different risk categories (high-risk - upper tertile; low-risk - lower 2/3 of cases of cases) on the basis of the coefficient computed in the logistic model. Two-tailed *P* values <0.05 were considered statistically significant. The Statistical Package performed statistical analyses for the Social Science SPSS/PC+ (20.0 edition). The permission to access the medical records was granted by the ethical committee of Romagna (CEROM), Italy (424/2019), considering the observational and retrospective nature of the study, conducted on anonymized records (Privacy guarantor act, GU 1st March 2012, n. 72).

## Results

The study population included 2,492 patients with diagnosis of sepsis: the mean age was 77.9 (SD 14.6), with 1,319 cases (52.9%) ≥80 years. The characteristics of patients in relation to age groups are summarized in Table 1. The proportion of men (total 1,355 (54.4%)) increased in relation to age groups: 48.2% in subjects <80 years vs. 51.8% in subjects **≥**80 (OR 1.10 95%CI 1.03 – 1.189; P=0.006). Over time, the number of subjects ≥80 doubled from 245 (2009-2010) to 484 (2015-2016), but no significant difference in percentage was observed (52.0%, 95%CI 47.4% - 56.4% (2009-2010) vs. 54.4% (95%CI 51.1% - 57.6%) (2015-2016); p = 0.876) (Table 1).

Main comorbidities were COPD (45.8%), cancer (42.0%), AHF (35.5%) CKD (33.6%), dementia (30.1%) and diabetes (29.2%), with COPD, diabetes, AHF, CKD, and dementia more represented in the group of subject ≥80 (Table 1). The SIRS score at entry in the ED was ≥ 2 in 75.4% of cases; ED waiting time was 60 min (SD 82): no differences between age groups were observed (Table 1).

The diagnosis of sepsis was suspected only in 32.9% of cases at admission (Table 1). The most common serious infection diagnoses occurred in the respiratory system (64.1%) and in the genitourinary tract (26.4%), with respiratory system infections more represented in subjects **≥**80 years (72.6% vs. 54.6%; OR 2.20 95%CI 1.86 – 2.60; P= 0.001) (Table 2). The organ/system dysfunction diagnoses more frequently observed were cardiovascular failure (27.1%), acute respiratory failure (57.8%) and renal failure (17.4%), cardiovascular failure and acute respiratory failure. These last two comorbidities were also more common in subjects ≥80 (30.0% vs. 24.0%, OR 1.36 95%CI 1.14 – 1.63; P <0.001, and 57.8% vs. 46.2% OR 1.60 95%CI 1.36 – 1.87; P=0.001, respectively) (Table 2).

Blood culture positive rates were recorded in 29.8% of total cases, with reduced rates in subjects **≥**80: anaerobic agents were most commonly represented (14.4%) (Additional file 1: Table S1). ED length of stay was 246 (SD 678) min and median hospital length of stay was 10 [IQR 14] days (6 [12] in subjects who died, 11 [12] in subjects discharged), without differences in relation to age groups.

The incidence rate (number of sepsis-related admissions per 100 total hospital admissions) increased from 3.8% (2009-2010) to 4.7% (2015-2016); *P* <0.001. This increase was especially observed in subjects ≥80 (Additional file 2: Table S2).

A total of 687 patients died (27.6%). Overall CFR decreased from 31.8% (95%CI 27.7% - 36.1%) (2009-2010) to 25.0% (95%CI 22.2% - 27.9%) (2015-2016) (*P* = 0.007), with a downward trend in the calendar year periods (P <0.003). We observed no difference in the percentage of subjects aged ≥80 who died: 52.9% (2009-2010) vs. 54.4% (2015-2016); *P* = 0.156.

**Fig. 1:**
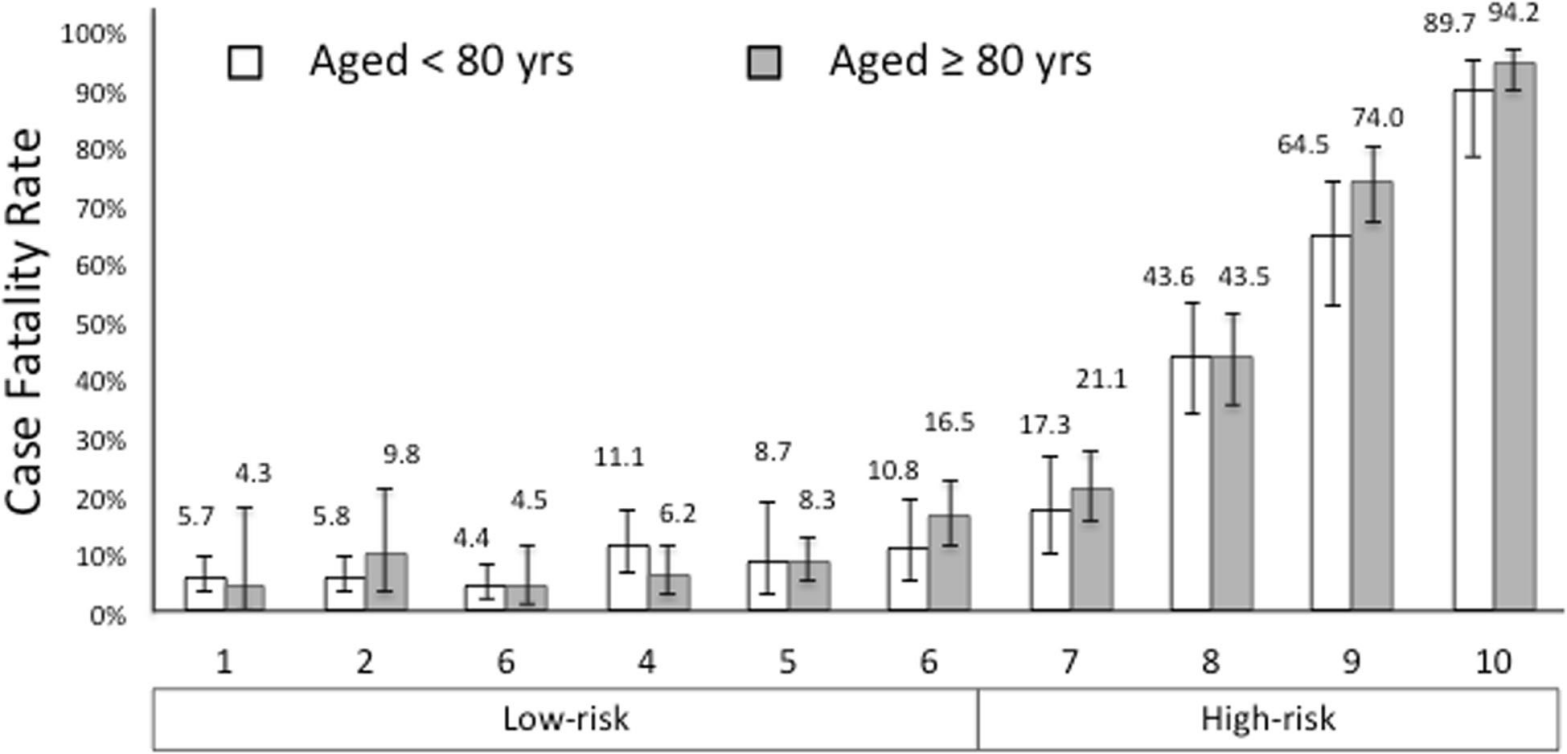
Proportion of case fatality rate (CFR) (median and 95% confidence intervals) by deciles of risk score and by age in subjects with sepsis related hospitalizations. Open columns represent CFR in subjects aged <80, grey columns are subjects ≥80

In the building model 10 items, out of the 27 tested, entered as outcome predictors (Table 3). Analytical and graphical methods showed that the proportionality assumption of the model was not violated (not reported in details) and the final model showed an overall accuracy (Area Under the Curve) of 0.848 ± SE 0.015; P <0.001. The overall accuracy of the model was confirmed in the validation dataset (Area Under the Curve) of 0.873 ± SE 0.009; P <0.001 (not different from the building cohort; DeLong test). The distribution of CFR according to risk score percentiles (deciles) in the entire population, grouped according to age, is reported in Fig. 1.

**Table 3:** Predictors of case fatality rate in subjects with sepsis related hospitalizations by variables included in the logistic model

Variables	Odds Ratio	95% CI	*P* value
ICU admission	15.03	7.33–30.81	<0.001
Cardio-vascular dysfunction	13.53	9.94–18.43	<0.001
HIV	10.94	3.75–31.93	<0.001
Diabetes	3.01	2.35–3.85	<0.001
Age ≥ 80 years	2.32	1.79–3.10	<0.001
Respiratory diseases	2.17	1.60–2.94	<0.001
Digestive diseases	1.93	1.27–2.93	0.002
Dementia	1.82	1.43–2.34	0.001
No. of organ dysfunction	1.62	1.27–2.06	<0.001
Cancer	1.48	1.17–1.88	0.001

Variables not included in the model: sex, mode of arrival in ED, the score criteria of Systemic Inflammatory Response syndrome (SIRS), infectious parasitic disease, nervous, circulatory and genitourinary diseases diagnoses, COPD, CKD, Charlson index, hematologic, neurologic, renal, respiratory and hepatic dysfunction, and ED waiting time, ED length-of-stay, as dichotomized variables. Data are reported as odds ratio and 95% confidence intervals (CI)

**Fig. 2:**
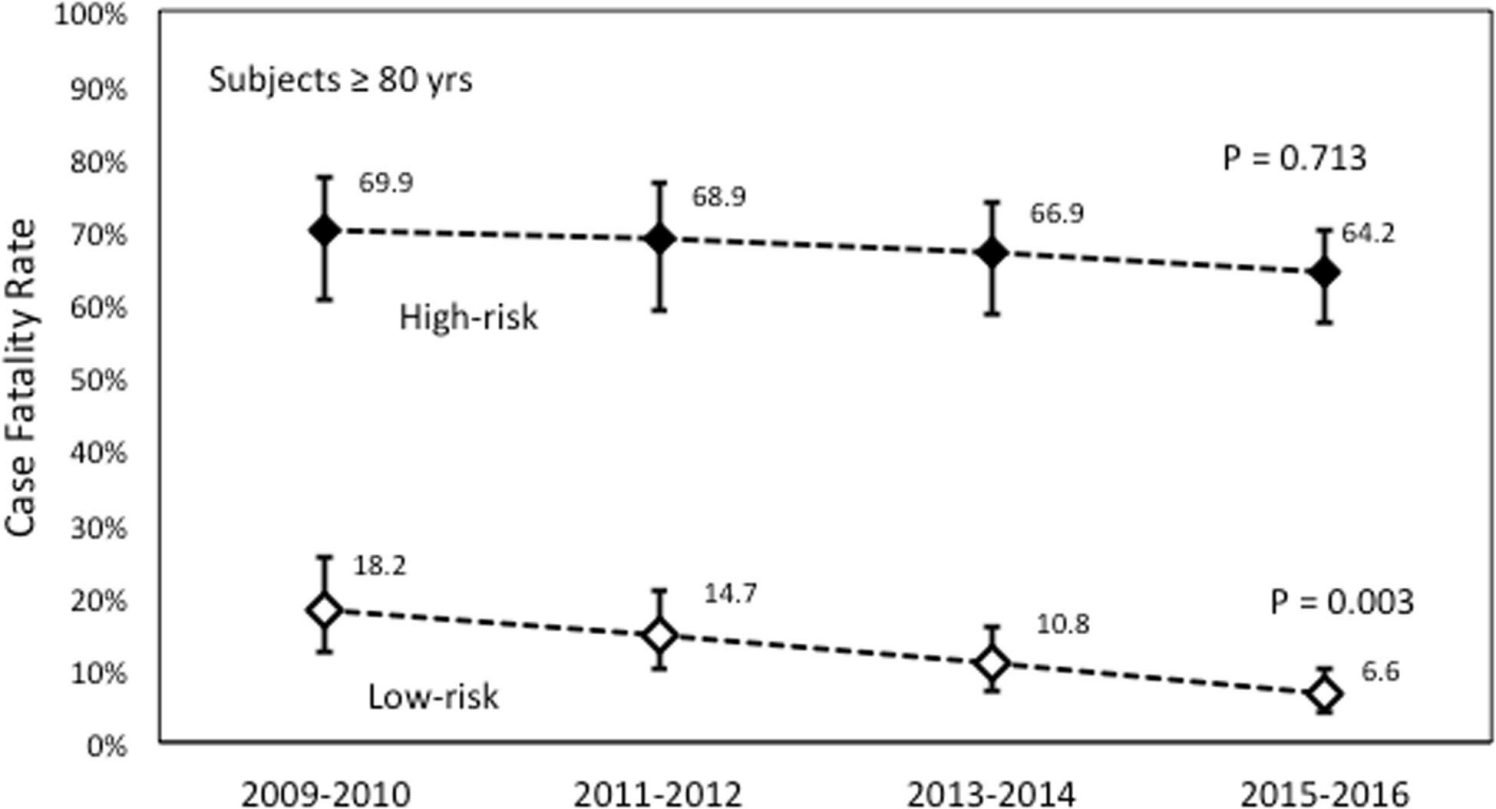
Temporal trends in case fatality rate (CFR) in high- (upper panel) and low-risk (lower panel) patients aged ≥80 with sepsis related hospitalizations Trends were tested by Poisson distribution analysis and represented by line graphs as mean with 95% confidence intervals in high-risk (full boxes) and low-risk (empty boxes) patients aged ≥80 with sepsis related hospitalizations.

When stratified by the risk score CFR decreased (from 13.3% (95%CI 9.8% - 17.4%) to 5.2% (95%CI 3.5% - 7.2%); *P* <0.001) in the low risk group. A progressive decline in CFR was confirmed both in the group of subjects **≥**80 years, where it decreased from 18.2% (95%CI 12.2% - 25.2%) to 6.6% (95%CI 4.1% - 10.0%) (*P*=0.003) (Fig. 2) and in the group of subjects aged <80 from 9.7% (95%CI 5.9% - 14.6%) to 3.9% (95%CI 2.1%- 6.4%) (*P*=0.010). In the total high-risk cohort, CFR was very high and did not change in the course of the years 66.9% (95%CI 59.0% - 73.3%) in 2009 and 62.5% (95%CI 56.8% - 67.6%) in 2016; *p* =0.743. This was also the case of subjects aged <80: 60.0% (95%CI 45.2% - 71.5%) to 58.9% (95%CI 48.4% - 67.8%); *P* = 0.972, and of subjects ≥80: 69.9% (60.4% - 77.2%) to 64.2% (95%CI 60.4% - 57.3% - 70.1%): *P* = 0.713 (Fig. 2)

## Discussion

The study provides three important messages: first, it confirms an increasing incidence of hospital admission with diagnosis of sepsis between 2009 and 2016; the incidence is largely driven by a more frequent occurrence in subjects ≥80 years; the CFR is declining, but this reduction is mainly limited to the elderly low-risk cohort. Finally, a group of 10 main predictors, i.e., older age, comorbidities, cardio-vascular dysfunction, number of organ dysfunctions and ED length-of stay predicted case fatality rate with good accuracy.

The incidence of sepsis has been reported to increase with age, mainly due to a sharp incidence in the group of subjects aged ≥80 [5], with high mortality rate [12–14]. In a recent large study nearly two thirds of patients admitted for sepsis were aged 65 or older [15] with age clearly associated with the development of sepsis. Our data probably reflect the peculiar Italian demography, with 84% of our patients over 65 and 53% over 80.

An increased incidence of cases with sepsis might be influenced by the combined effect of “up-coding diagnosis” and concurrent organ dysfunction diagnoses code. The first condition might stem from the diffusion of international guidelines of the surviving sepsis campaign [16], which leads to classify patients with serious infection as having sepsis; the second factor adds new diagnoses to the cases with diagnosis codes of sepsis/septic shock. Both effects are very likely to occur in low-risk cases and in older subject [17]. In our series, the number of sepsis diagnoses increased from 2009 to 2016, the relative percentage of low- vs. high-risk cases did not change over time, but mortality only decreased in low-risk patients. This finding is well in keeping with a possible effect of the surviving sepsis campaign, but also indicates that we could effectively improve the outcome only in less severe cases.

Our dataset captures all acute care hospitalizations for sepsis by ICD-9 codes. This method is currently accepted in sepsis epidemiology for assessing the main characteristics and trends for health care planning [2, 4]. Our study is based upon a hospital database, and the registration of organ dysfunction did not change over time; given the universalistic nature of the Italian health system, where coding practices are not conditioned by economic incentives, there is a low-risk of selection bias. The increased number of diagnosis code of sepsis is likely to stem from both increased awareness and knowledge among physicians, as well as a systematic up-coding driven by surviving sepsis campaigns [2].

Reduced mortality might also be associated with an increased up-coding effect, considering changes in diagnostic criteria (“serious infection associated with organ failure” adding to the sole “sepsis/septic shock”) [18, 19]. In our series we considered the novel criteria throughout the observation period, and the up-coding effect should be reduced to a minimum [18].

Considering the wide variability of the demographic and clinical characteristics of subjects with sepsis, several prognostic models were derived using different sets of variables. In a recent study a group of variables, i.e. age, the modified APACHE II score, ICU length of stay, patient location at sepsis diagnosis and coagulopathy were indicated as main outcome predictors [15]; in another study the selected variables entering the logistic model were only those associated with immediate fatality conditions, severity score or condition warranting intensive care admission and frailty, but not age [20, 21].

In our model, the area under the ROC curve (0.873) confirmed the validity of the logistic model, with selected variables indicating the patients’ clinical profile, serious infection and organ dysfunction diagnoses, not the early warning score for detection (SIRS score ≥ 2) at ED entry, which was positive in only 2/3 of cases. Other scores compete with SIRS as warning tools for the early detection of sepsis, but do not predict the mortality. Although not operative in our setting, we calculated a posteriori the qSOFA as another potential predictor of sepsis at entry [6]. The score, based on a combination of abnormal mental status, respiratory rate and systolic blood pressure, was positive (> 1) in 92.4% of cases, but did not enter the logistic regression when added to the model instead of SIRS, in keeping a higher sensitivity of qSOFA compared with SIRS [22].

The selected variables might be associated with different risk profile, in relation to the varying pattern of comorbidities in individual patients. This is definitely the case of the positive association between CFR and ICU admission, which is likely to be driven by severity and frailty. In our study mean age was 78 years and the comorbidities included in the logistic model were diabetes in 29%, cancer in 42%, dementia in 30%, HIV in a limited 1.0% of cases. Our case mix is very different compared with two recently published studies: in one study mean age was 67 years, with diabetes (35%), dementia (16%), cancer (24%) as principal comorbidities [23] and in another study mean age was 49 years, with diabetes present only in 12%, cancer in 14%), dementia in 0.4% [2].

Life sustaining treatment limitations in older subjects before the decision to admit patients to intensive care unit might further explain different results, since physicians might be reluctant to admit old patients to ICU despite proper admission criteria [24]. In our study patients admitted to ICU were 4%, with 1.5% over 80 years, and 1% over 90 years, a different result in comparison to 8.8% of cases, with only 0.4% over 90 years in a recent study [15]: this selection bias might explain part of inequalities of subjects included in different studies.In the last decade large epidemiological studies reported a downward trend of mortality in patients with sepsis, also in the elderly [25]. In a nationwide study in Taiwan the proportion of medical and surgical admissions for sepsis increased from 3.9% (2002) to 9.4% (2012) with in-hospital mortality rate decreasing from 24.1 to14.8% [5]; in detail, mortality rate decreased by 24% in subjects aged 65–84 and by 22% in the cohort of subjects > 84 years [2]. Such results were confirmed in a recent, retrospective, nation-wide Spanish study, where the percentage of hospital admissions due to sepsis increased from 3.6 to 5.8% and the case fatality rate decreased from 19.0 to 17.9%, with mortality rate highest in patients > 85 years. In these studies, in older subjects no risk score for disease severity was available and the increase in mortality was generally associated with high comorbidity rates, organ failure and high disease severity. In our series after stratification for the risk score, mortality rate over time decreased, and the downward trend in low-risk subjects occurred irrespective of the age cut-off of 80 years.

In a retrospective study a steadily increased mortality has been reported in middle-aged (45–64 years), compared to old (65–74 years) and very old ICU patients (> 75 years) [26], with rates increasing from 42.9 to 49.1% and to 56%, respectively. In our study CFR in subjects **≥**80 was as high as 35.6%, but the range was extremely wide in relation to the risk score. When stratified by the risk score, in the entire cohort CFR was as low as 11.2% in low-risk subjects (range, 9.1% - 13.%) compared to the high-risk individuals (68.6%, range 64.6–72.2%).

Limitations: first, although we included a comprehensive set of diagnostic codes of infectious disease to define sepsis, the incidence of sepsis in the elderly population might be nonetheless underestimated. Because of immune failure and functional decline, conventional clinical symptoms of inflammatory response may be lacking in older patients, or they might occur with atypical manifestations, like delirium or falls [27], which are frequent confounders for the rapid diagnosis of sepsis. Second, we used the all-cause mortality rather than sepsis-related mortality as primary endpoint of our study. A potential bias by indication may arise because older patients are also more likely to die for cardio-vascular and respiratory diseases. Third, a greater awareness of the putative severity of sepsis might drive an increased hospital admission. As with other metrics, if an increasing number of less sick patients is diagnosed with sepsis, CFR is expected to decrease [17]. Notably, only one third of cases were correctly classified as sepsis at entry in ED. Fourth, the analysis was based on a single center cohort, which may limit the external validity of the results (but increases the consistency of diagnostic procedures). Fifth, the study did not directly ascertain the sepsis mortality that might occur after hospital discharge. Finally, the severity of sepsis was not assessed by the appropriate SOFA (Sequential Organ Failure Assessment) score at entry [28], because of the retrospective nature of our chart review analysis and lack of important variables such as the time of onset of the clinical picture. On the other hand such approach made it possible to include consecutive patients, avoiding non-random selection.

## Conclusion

The incidence of hospital admission with diagnosis of sepsis is definitely increasing in the Italian healthcare system, with a downward trend in case fatality rate, also in very old subjects. This positive result, however, remains limited to low-risk subjects.

## Supplementary information

**Additional file 1: Table S1.** Characteristics and outcome measures of subjects with sepsis related hospitalizations from 2009 to 2016. Data are reported as number of cases (%).

**Additional file 2: Table S2.** Trends of incidence in 2492 subjects with sepsis related hospitalizations in the calendar periods between 2009 and 2016. Incidence is reported as number of events/100 admissions in the whole cohort and in different age groups.

## Data Availability

The datasets generated during and/or analyzed during the current study are available from the corresponding author on reasonable request.

## Abbreviations

AHF: History of acute heart failure
CFR: Case fatality rate
CKD: Chronic kidney disease
COPD: Chronic obstructive pulmonary disease
DM: Diabetes mellitus
ED: Emergency Department
ICU: Intensive care unit
qSOFA: quick Sequential Organ Failure Dysfunction
ROC: Receiver operating characteristic
SIRS: Systemic Inflammatory Response syndrome
