# Supplementary material for: Old subjects with sepsis in the emergency department: trend analysis of case fatality rate
Source: BMC Geriatr. 2019 Dec 23;19:372. doi: 10.1186/s12877-019-1384-8 (PMC6929475; doi:10.1186/s12877-019-1384-8)
Supplement: Supplementary file 1 — Additional file 1: Table S1. Characteristics and outcome measures of subjects with sepsis related hospitalizations from 2009 to 2016. Data are reported as number of cases (%). [file 12877_2019_1384_MOESM1_ESM.docx]

**Supplementary Table 1**

Characteristics and outcome measures of subjects with sepsis related hospitalizations from 2009 to 2016. Data are reported as number of cases (%).

| **Variables** | **All Cases** | **< 80 years** | **≥ 80 years** |
| --- | --- | --- | --- |
|  | (n = 2,492) | (n = 1,173) | (n = 1,319) |
| **Positive Blood Coltures** | 742 (29.8) | 455 (35.6) | 287 (23.7) |
| Anaerobic | 358 (14.4) | 217 (18.5) | 141 (10.7) |
| E. Coli | 84 (3.4) | 55 (4.7) | 29 (2.2) |
| Staphylococcus | 90 (3.6) | 65 (5.5) | 25 (1.9) |
| Not specified | 161 (6.5) | 85 (7.2) | 76 (5.8) |
| Others | 56 (2.2) | 40 (3.4) | 16 (1.2) |
| **Outcome measures** |  |  |  |
| Home | 1466 (58.8) | 798 (68.0) | 668 (50.6)* |
| In-hospital death | 687 (27.6) | 217 (18.5) | 470 (35.6)* |
| Non acute care facility | 179 (7.2) | 84 (7.2) | 95 (7.2) |
| Transfer to acute care hospital | 71 (2.8) | 44 (3.8) | 27 (2.0) |
| Hospice | 89 (3.6) | 30 (2.6) | 59 (4.5) |

* Significant difference vs. subjects <80 years, P <0.0
