# Supplementary material for: Old subjects with sepsis in the emergency department: trend analysis of case fatality rate
Source: BMC Geriatr. 2019 Dec 23;19:372. doi: 10.1186/s12877-019-1384-8 (PMC6929475; doi:10.1186/s12877-019-1384-8)
Supplement: Supplementary file 2 — Additional file 2: Table S2. Trends of incidence in 2492 subjects with sepsis related hospitalizations in the calendar periods between 2009 and 2016. Incidence is reported as number of events/100 admissions in the whole cohort and in different age groups. [file 12877_2019_1384_MOESM2_ESM.docx]

**Supplemental table 2:** Trends of incidence in 2,492 subjects with sepsis related hospitalizations in the calendar periods between 2009 and 2016. Incidence is reported as number of events/100 admissions in the whole cohort and in different age groups.

| **Variables** | **2009-2010** | **2011 - 2012** | **2013 - 2014** | **2015 - 2016** | **P Value** |
| --- | --- | --- | --- | --- | --- |
| **All cases** | 4.1 (3.8 – 4.5) | 5.0 (4.6 – 5.4) | 4.7 (4.3– 5.1) | 5.4 (5.1 – 5.8) | 0.007 |
| **Age ≥80 years** | 5.2 (4.6 – 5.8) | 5.3 (4.7 – 6.0) | 6.0 (5.4 – 6.6) | 6.5 (5.9 – 7.0) | 0.005 |
| **Age <80 years** | 3.4 (3.0 – 3.8) | 4.7 (4.1 – 5.2) | 3.7 (3.3 – 4.2) | 4.6 (4.1 – 5.0) | 0.006 |

Data reported as mean and (95% confidence interval)
